# Significance of Tumor Mutation Burden in Immune Infiltration and Prognosis in Cutaneous Melanoma

**DOI:** 10.3389/fonc.2020.573141

**Published:** 2020-09-18

**Authors:** Kai Kang, Fucun Xie, Jinzhu Mao, Yi Bai, Xiang Wang

**Affiliations:** ^1^Department of Medical Oncology, Peking Union Medical College Hospital, Chinese Academy of Medical Sciences, Beijing, China; ^2^Department of Liver Surgery, Peking Union Medical College Hospital, Chinese Academy of Medical Sciences, Beijing, China; ^3^Department of Hepatobiliary Surgery, First Central Hospital, Tianjin, China

**Keywords:** cutaneous melanoma, tumor mutation burden, immune infiltration, gene expression profile, functional enrichment analysis, prognosis, bioinformatics analysis

## Abstract

**Background:** Melanoma is highly immunogenic and therefore suitable for immunotherapy, but the efficacy is limited by response rate. In several types of tumor, tumor mutation burden (TMB) and immune infiltration have been reported to predict the response to immunotherapy, although each has its limitations. In the current study, we aimed to explore the association of TMB with immune infiltration and prognosis in cutaneous melanoma.

**Methods:** The data of cutaneous melanoma used for analyses was downloaded from The Cancer Genome Atlas (TCGA) database. The mutation data was sorted using “maftools” R package. TMB was estimated and then patients were divided into two groups based on TMB. The association of TMB with prognosis and clinical characteristics was explored. Differential analysis between two TMB groups was performed using “DESeq2” R package to identify differentially expressed genes (DEGs). The function enrichment analyses of DEGs were conducted to screen critical pathways. Besides, DEGs were further filtered to identify two hub genes, based on which a risk score model and nomogram for predicting prognosis were conducted, and the validation was performed using three datasets from Gene Expression Omnibus (GEO) database. Finally, CIBERSORT algorithm and TIMER database were used to assess the effect of TMB and hub genes on immune infiltration.

**Results:** The most common mutation was C > T, and the top three frequently mutated genes were *TTN, MUC16*, and *BRAF*. Higher TMB indicated better survival outcomes and lower pathological stages. 735 DEGs were identified and mainly involved in immune-related and adhesion-related pathways. The risk score model and nomogram were validated using receiver operating characteristic (ROC) curves and calibration curves, and exhibited relatively high predictive capability. Decision curve analysis (DCA) was used to assess clinical benefit. As for immune infiltration, the proportion was higher for macrophages M1 and M2 in the high-TMB group, while lower for memory B cells and regulatory T cells.

**Conclusions:** In cutaneous melanoma, TMB was positively correlated with prognosis. The risk score model and nomogram can be conveniently used to predict prognosis. The association of TMB with immune infiltration can help improve the predicting methods for the response to immunotherapy.

## Introduction

Melanoma is a malignant tumor derived from pigment-producing melanocytes (1). In the past decades, the incidence of melanoma has increased rapidly (2, 3). In the United States, melanoma is now estimated as the fifth most common cancer, and the probability of developing melanoma in a lifetime is 1 in 63 (4, 5). Early-stage melanomas have good prognosis after surgery, but even relatively small melanomas have metastatic potential because of the loss of cellular adhesion (6–8). Prior to 2011, chemotherapy was regarded as the standard treatment for metastatic melanoma, with a 5-year survival rate of 15.7% (9). Fortunately, melanoma is one of the most immunogenic tumors and therefore has the greatest potential for response to immunotherapy (10), so that it has been the most important tumor driving the development of solid tumor immunotherapy, especially immune checkpoint inhibitors (ICIs) targeting such as programmed cell death protein 1 (PD-1), PD-1 ligand (PD-L1), and cytotoxic T lymphocyte antigen 4 (CTLA4) (11). ICIs have greatly improved the survival of patients with advanced melanoma. In recent clinical trials, the 5-year survival rate treated with pembrolizumab and combination of nivolumab and ipilimumab in advanced melanoma was 34 and 52%, respectively (12, 13).

Considering the different therapeutic efficacy among patients, it is necessary to predict the response to immunotherapy. Although PD-L1 immunohistochemistry is the most widely used test to estimate the treatment response to ICIs, it has no influence on the treatment decision in most cases (14, 15). Since melanocytes are usually exposed to a large amount of ultraviolet radiation and the accumulated mutations, melanomas have a higher mutational load than other tumors (16), which may increase the efficacy of ICIs by generating and presenting immunogenic neoantigens (17, 18). Studies have found that predicting the response to immunotherapy through the overall mutational load may serve as a new perspective (19, 20).

Tumor mutation burden (TMB), defined as the total number of somatic coding errors, base substitutions, and indel mutations per million bases (21), can effectively estimate overall mutational load and neoantigenic load (22). Besides, many studies discovered that TMB can be used as a biomarker to predict the response to immunotherapy and the efficacy of ICIs in many cancer types including melanoma (23–27). However, few studies have focused on the TMB-related immune cell infiltration and gene signature in melanoma, so we conducted the current study to explore the prognostic role of TMB and the association with immune infiltration and gene signature in melanoma.

In the current study, based on the data of cutaneous melanoma from The Cancer Genome Atlas (TCGA) database, we explored the correlation between TMB and prognosis, differentially expressed genes (DEGs) between high- and low-TMB groups, the functional enrichment of DEGs, and the association of TMB with immune infiltration. Additionally, we constructed a risk score model according to TMB-related gene signature, completed the verification in three Gene Expression Omnibus (GEO) datasets, and developed a nomogram in combination with clinical characteristics.

## Methods

### Data Source and Mutation Analysis

Somatic mutation data in the “Masked Somatic Mutation” type processed by VarScan2 (28), transcriptome profiles in HTseq-Counts workflow type, and clinical data of skin cutaneous melanoma (SKCM) patients were downloaded from TCGA-SKCM project in TCGA database (https://portal.gdc.cancer.gov/). Mutation analysis, the first step of process, was conducted based on all available somatic mutation data of patients without exclusion. Subsequent analyses were based on transcriptome profiles and clinical data, so patients with incomplete information and zero survival time were excluded. Besides, the transcriptome profiles and clinical data of validation sets were obtained from the GEO database (http://www.ncbi.nlm.nih.gov/geo/). Subsequently, we visualized the somatic mutation data in Mutation Annotation Format (MAF) using the “maftoools” R package, which provides a large amount of commonly used analysis and visualization modules in cancer genomic studies (29).

### TMB Value Estimation and Prognostic Analysis

We extracted the somatic mutation information through a Perl script, after which TMB value can be estimated through dividing the number of somatic mutations by the total length of exons. Then we utilized R to merge the patient's TMB information and clinical information, including survival time and survival status. The optimal cutoff value of TMB was determined using maximally selected rank statistics from the “maxstat” R package, which is an outcome-oriented method providing the cut-point that correspond to the most significant relation with survival. Each TMB value was taken in turn as the cutoff value to find the situation with the most significant difference in survival between two groups. After dividing the patients into high- and low-TMB groups based on the optimal cutoff value, we performed Kaplan–Meier (K–M) survival analysis and log-rank test to compare the difference of overall survival (OS) between the above two TMB groups. Additionally, we explored the relationship between TMB and several clinical features including age, gender, pathological stage, and American Joint Committee on Cancer (AJCC) TNM staging. Wilcoxon rank-sum test was employed if patients were divided into two groups based on the clinical feature, while the Kruskal–Wallis test was used for more than two groups.

### Identification of DEGs

According to the above groups based on TMB, we performed normalization and differential gene expression analysis using the “DESeq2” R package. The normalization was based on the “Relative Log Expression” method, which is specifically implemented in the “DESeq2.” The scaling factors were calculated using the median ratio between gene abundances and the geometric mean. As a method for differential analysis of transcriptome count data, DESeq2 improves the interpretability and stability of estimation because of shrinkage estimators for fold change (FC) and dispersion (30). The differential gene expression analysis was conducted. Then we specified |log_2_FC| > 1.5 and false discovery rate (FDR) < 0.05 as cutoffs to identify qualified DEGs for subsequent analyses, and generated heatmap using the “pheatmap” R package.

### Functional Enrichment Analysis

After obtaining the Entrez-ID of each DEG through the “org.Hs.eg.db” R package, we performed Gene Ontology (GO) and Kyoto Encyclopedia of Genes and Genomes (KEGG) pathway analyses and visualized the results, using the “clusterProfiler,” “enrichplot,” and “ggplot2” R packages (31). The interactions between significant KEGG pathways were further visualized by Cytoscape software (version 3.8.0) (32). In addition, gene set enrichment analysis (GSEA), which is not restricted by DEGs, was used to understand TMB-related pathways. With GSEA software (version 4.0.3) (http://software.broadinstitute.org/gsea/index.jsp) (33), we utilized TMB as a phenotype label and chose the gene set named “c2.cp.kegg.v7.1.symbols.gmt” from Molecular Signature Database as the reference.

### Protein-Protein Interaction Network

Based on the STRING database (https://string-db.org/) (34), the protein-protein interaction (PPI) network of DEGs was constructed, followed by importing the results into Cytoscape software. Cytohubba plugin was used to rank nodes and identify hub objects from the complex network by “degree ranking method” (35). Molecular Complex Detection (MCODE) plugin was used to detect densely connected regions and identify clusters in the network (36). Finally, we performed functional enrichment analyses on the subnetworks obtained from MCODE.

### Construction and Verification of Risk Score Model

A list of 1,811 immune-related genes was downloaded from the Immunology Database and Analysis Portal (ImmPort) database (https://www.immport.org/shared/genelists/) (37, 38), followed by intersecting the list with DEGs and further visualization via “VennDiagram” R package. Based on the expression level of differentially expressed immune genes, batch survival analysis was performed to evaluate the relationship between gene expression after log_2_(count+1) transformation and OS of melanoma patients. Then the significant candidate genes were further filtrated via least absolute shrinkage and selector operation (LASSO) and stepwise regression. We developed a risk score model using the product of the mRNA level of qualified hub genes and respective regression coefficients. Subsequently, we performed K–M analysis based on the risk score and receiver operating characteristic (ROC) curve via the “survivalROC” R package to evaluate the performance of the model.

As for external validation, according to the filter criteria as: (1) patients had been diagnosed as melanoma, (2) the datasets include complete survival information, and (3) include enough sample sizes (*n* > 50), three melanoma datasets were chosen, GSE65904 (*n* = 210), GSE54467 (*n* = 79), and GSE22153 (*n* = 54) as validation sets in the GEO database. We conducted log_2_(count+1) transformation of the gene expression data, took the average value when duplicate data was found, and then verified the predictive accuracy of the risk score model using the ROC curve and K–M analysis.

### Development and Evaluation of the Nomogram

Through univariate Cox regression analysis, we evaluated the significance of prognostic risk score and clinical features to predict survival outcomes, using *P* < 0.05 as the cutoff. Then significant factors were further assessed by multivariate Cox regression analysis to exclude confounding factors, followed by performing the nomogram via the “rms” R package. To evaluate the predictive accuracy of the nomogram in TCGA cohort, we calculated Harrell's concordance index (C-index) using the “survival” R package to quantify the discrimination performance, and plotted calibration curves of survival probability at different years via Hosmer–Lemeshow test. ROC curves were performed to evaluate the accuracy of the nomogram. In addition, decision curve analysis (DCA) was conducted to assess the clinical outcomes of different decision strategies (39).

### Evaluation of Immune Cell Infiltration

As a versatile deconvolution algorithm for quantifying cell fractions of complex tissues from gene expression profiles (40), the CIBERSORT (R scrip v 1.03), with leukocyte signature matrix termed LM22 as a template, can calculate the distribution of 22 types infiltrating immune cells based on the transcriptome profiles (41). After calculation and filtration with *P* < 0.05, the proportions of different immune cells in each melanoma sample were exhibited via barplot function. As for the association with TMB level, Wilcoxon rank-sum test was used to compare the differences in the content of each type of immune cells between two TMB groups, and the results were visualized using the “vioplot” R package.

Tumor Immune Estimation Resource (TIMER) web server (https://cistrome.shinyapps.io/timer/) pre-calculated the abundance of six tumor-infiltrating immune subsets, including B cells, CD4+ T cells, CD8+ T cells, macrophages, neutrophils, and dendritic cells, in samples across 32 cancer types from TCGA (42). The modules in TIMER were used to explore the association of immune infiltration with gene expression and survival outcomes in the current study. In melanoma, for each hub gene involved in the risk score model, Somatic Copy Number Alterations (SCNA) module of TIMER tool was used to compare the infiltration levels among samples with different SCNA, including deep deletion, arm-level deletion, diploid/normal, arm-level gain, and high amplification (43). Furthermore, we explored the relationship between six tumor-infiltrating immune subsets and OS. Using the Survival module of TIMER, a Cox regression model was constructed based on the abundance of six immune cells in melanoma.

### Statistical Analysis

R software (version 4.0.0) was used for statistical analyses, and the R packages used in each step are mentioned above. The R codes involved in this study could be downloaded from the link https://github.com/kkang97/TMB-melanoma. Survival analyses were performed by the K–M method and the log-rank test. Univariate and multivariate Cox regression analyses were used to evaluate the significance of prognostic factors. Wilcoxon rank-sum test and Kruskal–Wallis test were used for subgroup differential analyses. All statistical tests were two-sided, and *P* < 0.05 was considered statistically significant.

## Results

### Mutation Analysis

**Figure 1:**
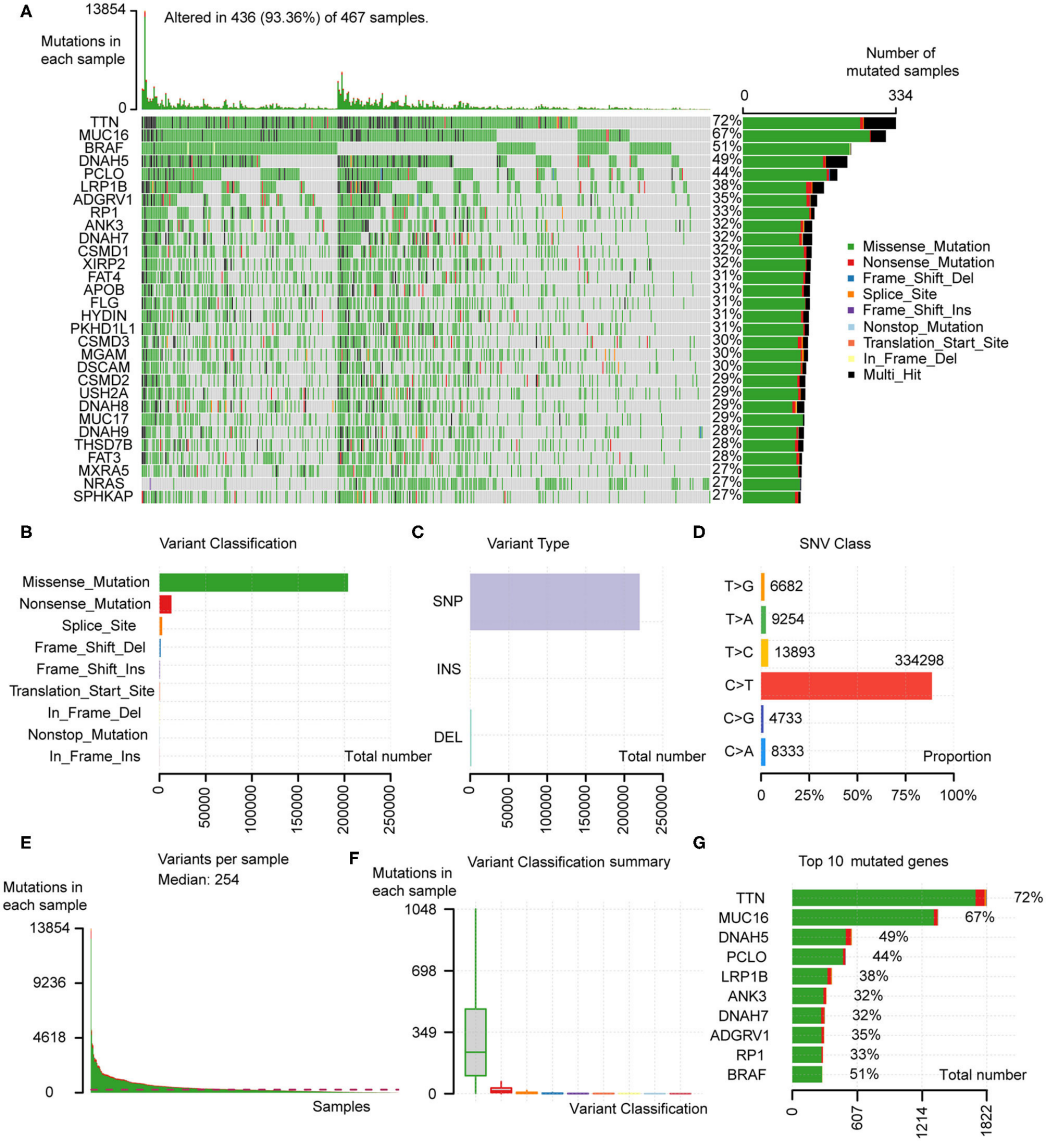
Analyses of somatic mutation profiles in melanoma samples. **(A)** Waterfall plot of detailed mutation information of top 30 genes in each sample, with various color annotations to distinguish different mutation types. **(B–D)** According to different classification categories, missense mutation, SNP, and C > T mutation accounted for the overwhelming majority. **(E)** The total mutation number in each sample. **(F)** Box plots of each variant classification in each sample. **(G)** Top 10 mutated genes in melanoma. SNP, single nucleotide polymorphism; SNV, single nucleotide variants.

Somatic mutation profiles of 467 melanoma patients downloaded from TCGA database were analyzed and visualized via the “maftools” R package. The waterfall plot was performed to exhibit the detailed mutation information in each sample, with various color annotations to distinguish different mutation types (Figure 1A). According to further comparison, missense mutations, single-nucleotide polymorphism (SNP), and C > T mutation accounted for the vast majority of different classification categories, respectively (Figures 1B–D). Counting each sample separately, the median of mutations in the sample was 254, and the maximum was 13,854 (Figure 1E). In addition, we exhibited the number of each variant classification in the different sample via box plots (Figure 1F). The top 10 mutated genes in 467 melanoma patients are *TTN* (72%), *MUC16* (67%), *BRAF* (51%), *DNAH5* (49%), *PCLO* (44%), *LRP1B* (38%), *ADGRV1* (35%), *RP1* (33%), *ANK3* (32%), *DNAH7* (32%) (Figure 1A). Moreover, counting the multiple hits separately and considering the total number of mutations, the top 10 genes were different from the previous ones (Figure 1G).

### Correlation of TMB With Prognosis and Clinical Features

**Figure 2:**
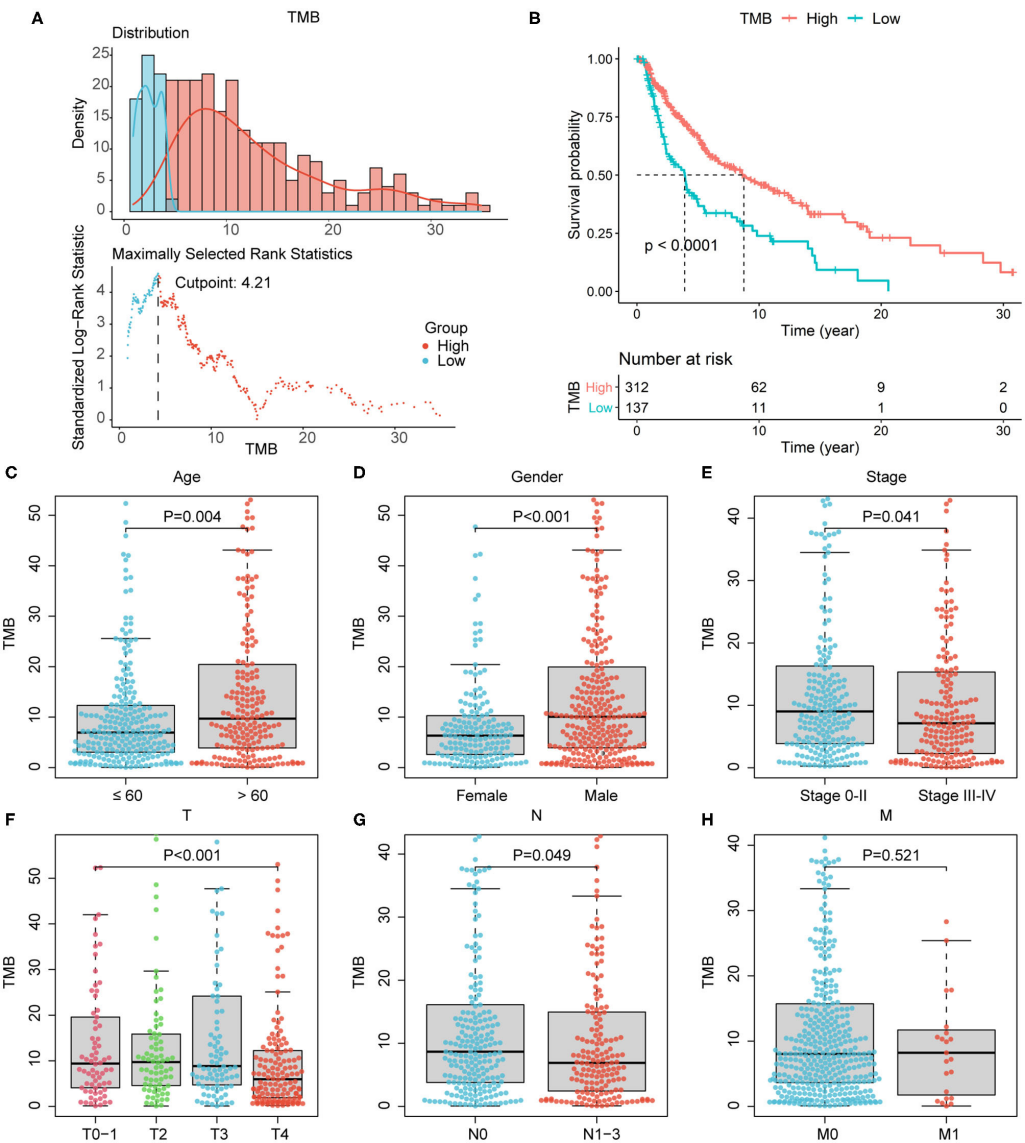
Association of TMB with prognosis and clinical features. **(A)** The optimal cutoff of 4.22 according to maximally selected rank statistics. **(B)** According to survival analysis, high-TMB group correlated with better survival outcomes, with *P* < 0.0001. **(C–G)** Age over 60 years old, male, lower pathological stage, lower AJCC-T, and N stage correlated with higher TMB level. **(H)** The association of TMB with AJCC-M stage was not significant. AJCC, American Joint Committee on Cancer.

Since the data of somatic mutation, transcriptome profiles, and clinical information were collected from highly overlapping melanoma patient population, we selected 449 patients with complete information above for subsequent analyses (Table 1), in order to improve the credibility of the conclusions with only a little cost of data loss. After estimated TMB, we calculated the optimal cutoff value of 4.22 based on maximally selected rank statistics, and divided 449 patients into high- (*n* = 312) and low-TMB groups (*n* = 137; Figure 2A). According to the K–M curve, the high-TMB group had significantly better survival outcomes, with the log-rank test of *P* < 0.0001 (Figure 2B). Besides, we explored the correlation of TMB with clinical features and revealed age, gender, pathological stage, AJCC-T, and N stage, were significantly associated with TMB (Figures 2C–H). Age over 60 years old, male, lower pathological stage, AJCC-T, and N stage represented a higher TMB level.

**Table 1:** Clinical baseline of 449 melanoma patients in TCGA cohort.

**Variables**	**Number (%)**
**Status**	
Alive	234 (52.12%)
Dead	215 (47.88%)
**Age**	57.9 ± 15.6
**Gender**	
Male	280 (62.36%)
Female	169 (37.64%)
**Pathological stage**	
Stage 0	6 (1.34%)
Stage I	85 (18.93%)
Stage II	133 (29.62%)
Stage III	168 (37.42%)
Stage IV	22 (4.90%)
Unknown	35 (7.80%)
**AJCC-T stage**	
T0/Tis	30 (6.68%)
T1	40 (8.91%)
T2	76 (16.93%)
T3	88 (19.60%)
T4	144 (32.07%)
Unknown	71 (15.81%)
**AJCC-N stage**	
N0	221 (49.22%)
N1	73 (16.26%)
N2	48 (10.69%)
N3	54 (12.03%)
Unknown	53 (11.80%)
**AJCC-M stage**	
M0	400 (89.09%)
M1	23 (5.12%)
Unknown	26 (5.79%)
**Sample type**	
Primary tumor	97 (21.60%)
Satellite and in-transit metastasis	70 (15.59%)
Regional lymph node	215 (47.88%)
Distant metastasis	64 (14.25%)
Unknown	3 (0.67%)
**TMB**	
High level	312 (69.49%)
Low level	137 (30.51%)

### Functional Enrichment Analysis and PPI Network

**Figure 3:**
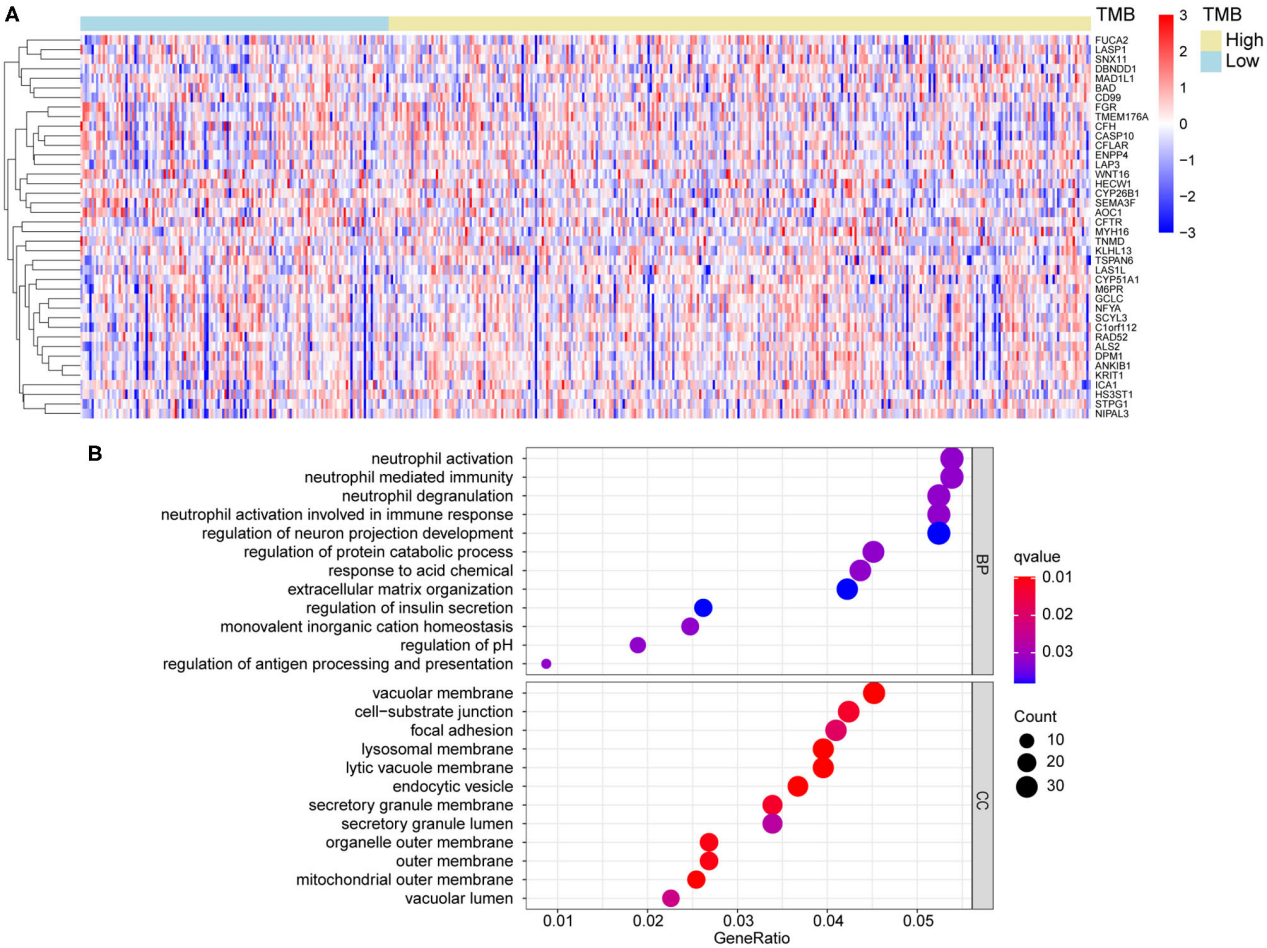
Differential gene analysis and GO analysis. **(A)** Heatmap of top 40 DEGs between high- and low-TMB groups. **(B)** DEGs were mainly enriched in immune related and cell adhesion related pathways. DEGs, differentially expression genes. BP, biological process; CC, cellular component.

**Figure 4:**
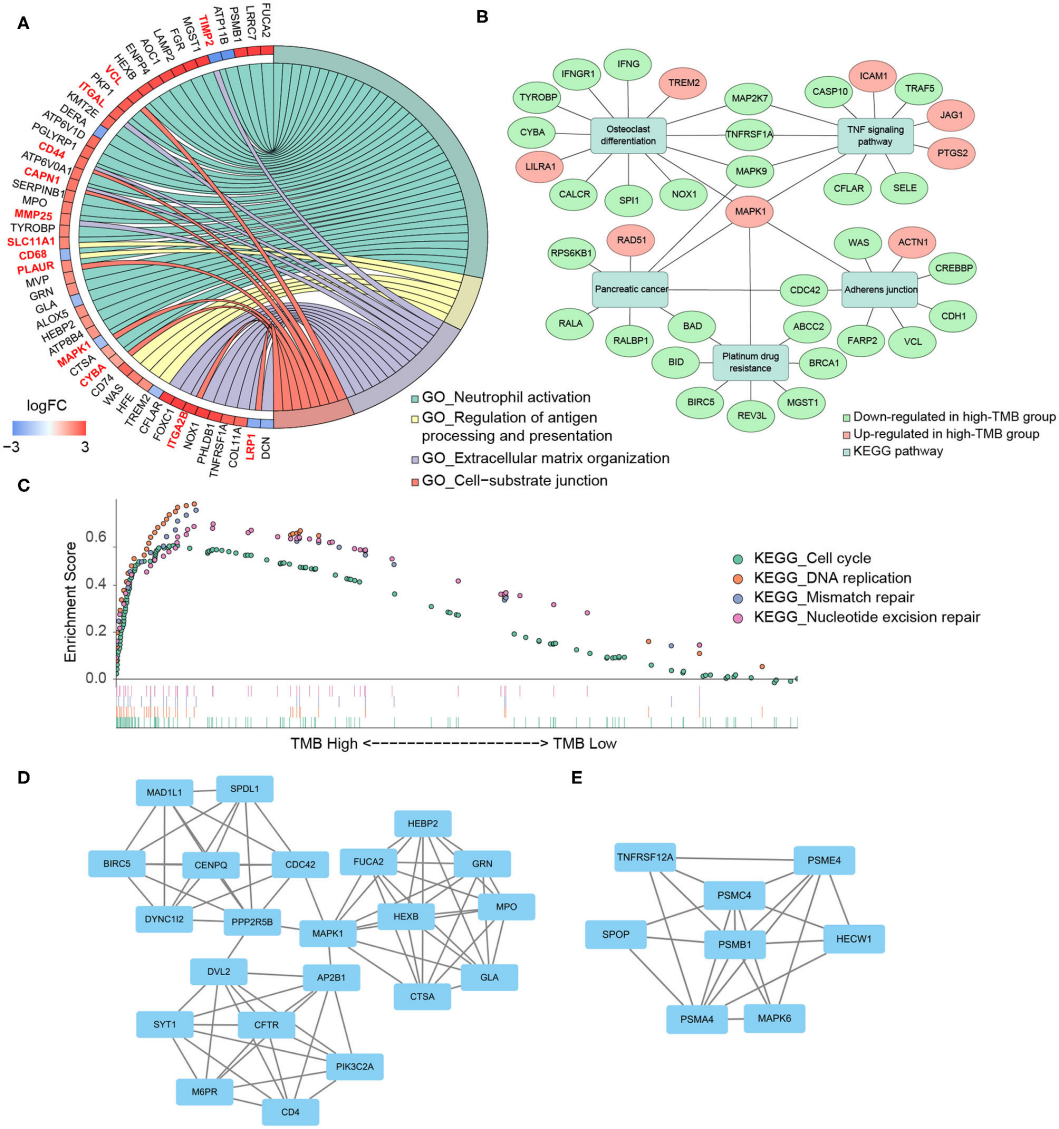
Functional enrichment analysis and PPI subnetworks. **(A)** Chord diagram further exhibited the relationship between DEGs and GO pathways. **(B)** MAPK1 gene played a critical role in multiple significant KEGG pathways. **(C)** TMB-related pathways, explored by GSEA, including cell cycle, DNA replication, mismatch repair, and nucleotide excision repair, with FDR < 0.025. **(D,E)** Two significant subnetworks of PPI network.

According to the differential gene expression analysis, in which high-TMB group was set as treatment group, while low-TMB group as control group, a list of 735 DEGs with |log_2_FC| > 1.5 and FDR <0.05 was identified, including 183 genes up-regulated and 552 genes down-regulated in the high-TMB group (Supplementary Table 1). Top 40 DEGs ranked in the order of FDR were visualized in the heatmap (Figure 3A). According to GO analysis, DEGs were mainly enriched in immune-related pathways such as neutrophil activation, neutrophil degranulation, and cell adhesion-related pathways such as cell-substrate junction, focal adhesion (Figure 3B). From each type of pathways, we selected representative ones to perform a chord diagram, making the connection between pathways more intuitive (Figure 4A). Besides, we performed KEGG pathway analysis based on DEGs (Supplementary Figure 1). Then we selected several significant pathways of interest from the results, such as osteoclast differentiation, tumor necrosis factor (TNF) signaling pathway, and adherens junction, for subsequent visualization by Cytoscape, and found the *MAPK1* gene was involved in multiple significant KEGG enrichment pathways (Figure 4B). Furthermore, we explored the TMB-related pathway through GSEA, using TMB level as the phenotype label, and found that cell cycle, DNA replication, mismatch repair, and nucleotide excision were significantly enriched in the high-TMB group, with FDR < 0.025 (Figure 4C).

Through the STRING database, we set the minimum required interaction score as 0.7 and then constructed a PPI network of DEGs (Supplementary Figure 2). Important node genes and subnetworks were further analyzed by Cytohubba and MCODE plugins, respectively. Ranked by the degree method of Cytohubba, the top 10 significant node genes were *CDC42, MAPK1, POLR2B, POLR2J, CUL3, CDC27, RNF4, FBXW11, KLHL13*, and *BRCA1*. Meanwhile, we used MCODE to detect densely connected regions and identify two significant subnetworks (Figures 4D,E). GO analyses of these two subnetworks revealed the function of the first subnetwork was enriched in neutrophil activation and degranulation, while the function of the second subnetwork was enriched in tumor necrosis factor-mediated signaling pathway, regulation of Wnt signaling pathway, and antigen processing and presentation (Table 2, Supplementary Table 2).

**Table 2:** Representative GO results of two subnetworks.

**ID**	**Description**	**Count**	***p*-value**	***q-*value**
**Subnetwork 1**				
GO:0043312	Neutrophil degranulation	8	4.55E-08	1.11E-05
GO:0042119	Neutrophil activation	8	5.58E-08	1.11E-05
GO:0007059	Chromosome segregation	5	3.02E-05	4.75E-03
**Subnetwork 2**				
GO:0033209	Tumor necrosis factor-mediated signaling pathway	5	2.96E-09	8.86E-08
GO:0030111	Regulation of Wnt signaling pathway	5	1.44E-07	2.69E-07
GO:0019882	Antigen processing and presentation	4	1.41E-06	1.53E-06

### Risk Score Model and Nomogram

**Figure 5:**
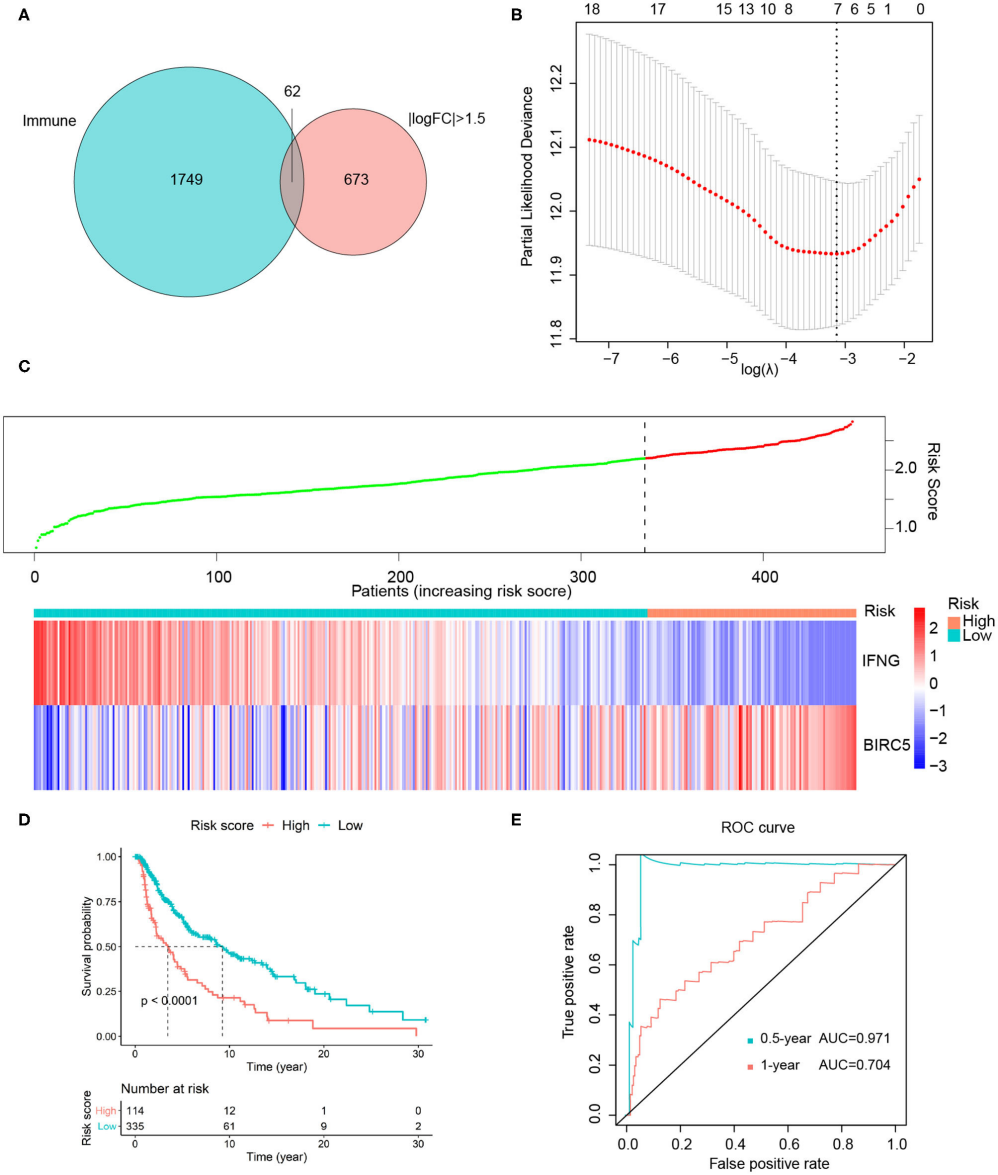
Construction of risk score model. **(A)** Intersection of 735 DEGs and 1811 immune-related genes. **(B)** Seven genes were identified from 18 candidate genes using LASSO regression. **(C)** The distribution of risk score and gene expression levels among patients. **(D)** High-risk group Correlated with poor survival outcome, with *P* < 0.0001. **(E)** ROC curves of 0.5- and 1-year survival prediction, with AUC = 0.971 and 0.704, respectively. FC, fold change.

Due to the high immunogenicity of melanoma and the relationship between TMB and immune pathways, we considered establishing the risk score model based on 62 differentially expressed immune genes, namely the intersection of 735 DEGs and 1,811 immune-related genes (Figure 5A). To screen prognostic hub genes, we utilized batch survival K–M analysis on candidate genes, with *P* < 0.001 as the cutoff. Then LASSO regression was performed on the remaining 18 genes for further filtration, and seven genes were identified for subsequent analysis (Figure 5B). Finally, through stepwise regression, two immune-related DEGs (*IFNG* and *BIRC5*) were selected as prognostic modeling genes. The risk score model was constructed as follows: *risk score*** = **−0.13914^*^IFNG expression level + 0.21596^*^BIRC5 *expression level*. The positive coefficient of *BIRC5* in the formula represented that its high expression indicated poor survival outcomes, while the negative coefficient of *IFNG* implied an opposite association. And similar conclusions were obtained by previous survival analyses of these two genes (Supplementary Figures 3A,B). Besides, the distribution of risk score and the gene expression levels in patients were analyzed and exhibited (Figure 5C).

**Figure 6:**
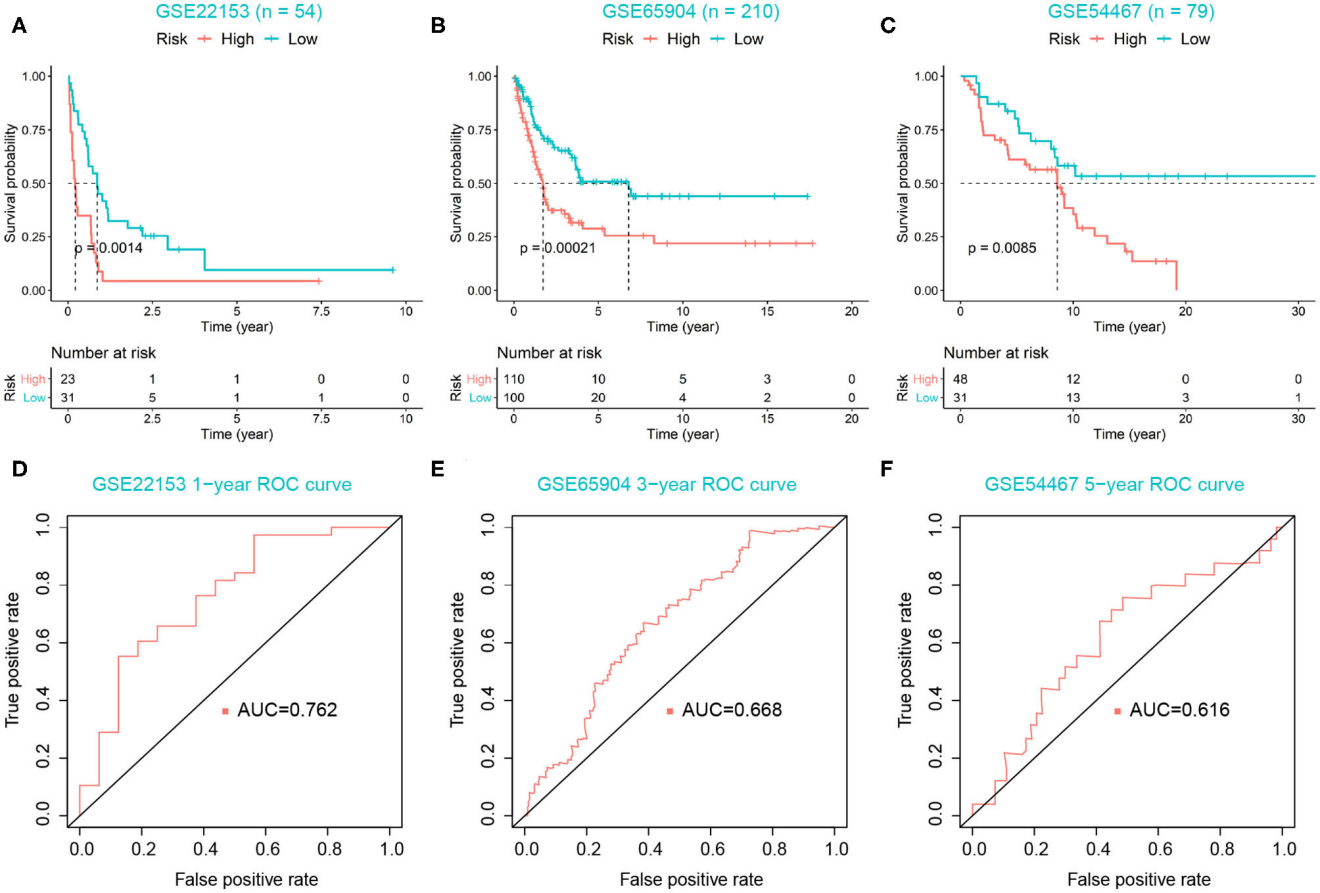
Validation of risk score model. **(A–C)** High-risk group correlated with poor survival outcomes in three validation sets, with *P* = 0.0014, 0.00021, 0.0085, respectively. **(D–F)** ROC curves were used to evaluate the predictive accuracy of the risk score model in three validation sets.

For 449 cutaneous melanoma patients from the TCGA database, we calculated the risk score and identified the optimal cutoff by maximally selected rank statistics, and divided the patients into high- (*n* = 114) and low-risk (*n* = 335) groups. The K–M analysis exhibited the survival outcomes of the high-risk group were significantly worse, with *P* < 0.0001 (Figure 5D). Meanwhile, we used the ROC curves of 0.5 and 1-year OS prediction based on risk score to evaluate the predictive accuracy, with the area under the curve (AUC) = 0.971 and 0.704, respectively (Figure 5E). As for external validation, GSE22153, GSE65904, and GSE54467 were chosen as validation sets. The K–M analysis exhibited a significant shorter OS of high-risk group in each validation set, with *P* = 0.0014, 0.00021, 0.0085, respectively (Figures 6A–C). According to the different range of OS in each validation set, ROC curves were performed for 1-, 3-, and 5-year OS prediction, respectively, and the AUCs were 0.762, 0.668, and 0.616 (Figures 6D–F).

**Figure 7:**
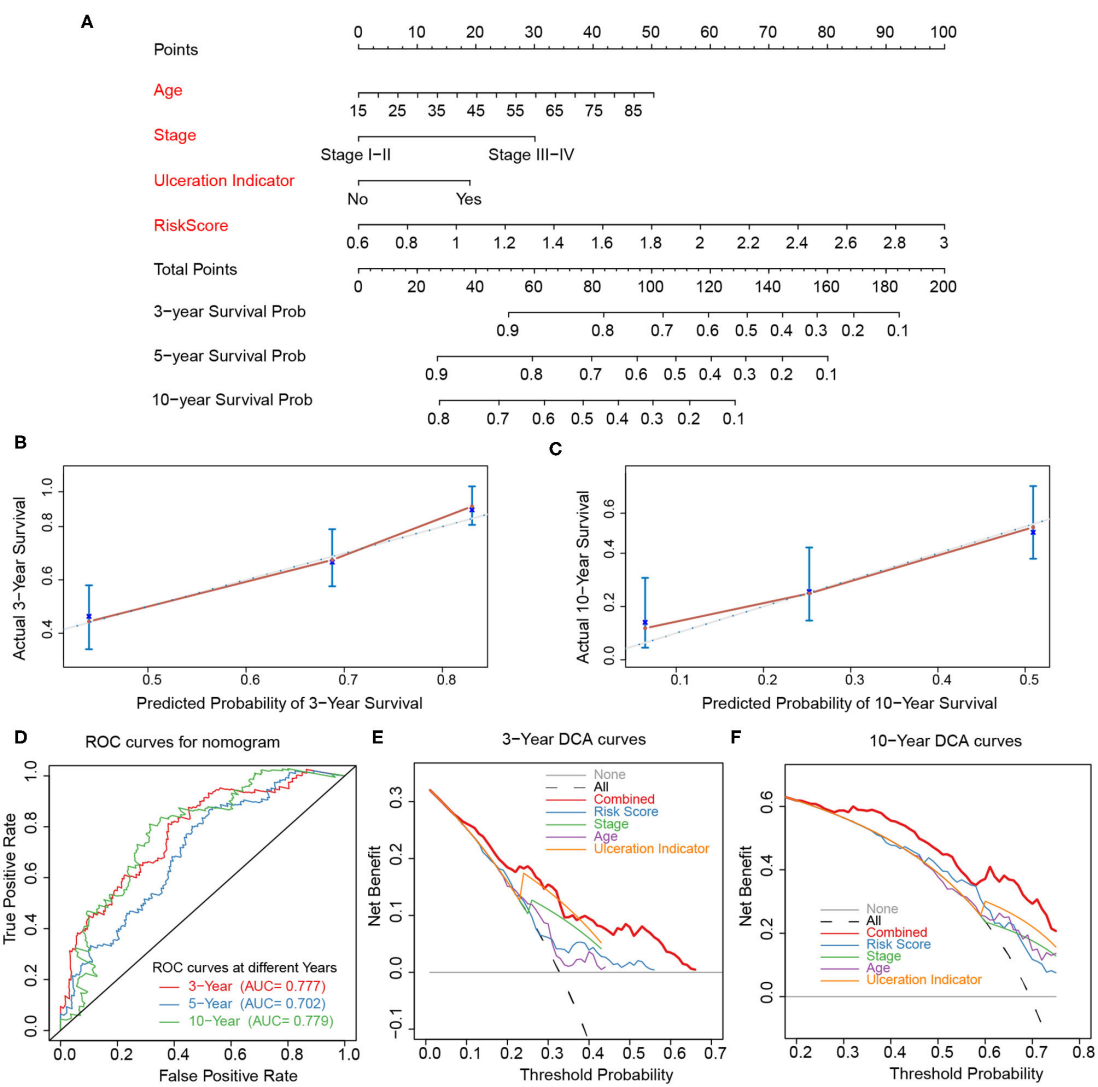
Construction and validation of nomogram. **(A)** Nomogram constructed based on age, stage, ulceration indicator, and risk score as predictive factors to predict 3-, 5-, 10-year survival probability. **(B,C)** Calibration curves for the survival probability at 3 and 10 years. **(D)** ROC curves for 3-, 5-, 10-year prediction of the nomogram. **(E,F)** DCA curves to evaluate the clinical utility of different decision strategies, and the red line represented the combined nomogram.

The risk score, age, pathological stage, and ulceration indicator were screened as significant predictive factors by univariate and multivariate Cox regression analyses (Table 3). A nomogram was performed based on the above predictive factors (Figure 7A). The C-index was 0.702, and its standard error was 0.024. The calibration curves for the survival possibility at 3 and 10 years exhibited the accurate prediction ability of nomogram in both short- and long-term (Figures 7B,C). The AUCs of ROC curves were 0.777, 0.702, 0.779 for 3-, 5-, and 10-year OS prediction, respectively (Figure 7D). The DCA exhibited a higher net benefit of decision based on nomogram compared to individual predictive factors (Figures 7E,F).

**Table 3:** Univariate and multivariate Cox regression analyses of clinical features and risk score with OS.

**Variables**	**Univariate analysis**			**Multivariate analysis**		
	**HR**	**95% CI of HR**	***P-*value**	**HR**	**95% CI of HR**	***P-*value**
Gender (Male/Female)	1.102	0.829–1.466	0.502			
Age	1.024	1.015–1.034	<0.001	1.016	1.004–1.028	0.010
Pathological stage (III–IV/I–II)	1.630	1.216–2.184	0.001	2.006	1.414–2.847	<0.001
Ulceration indicator (Yes/No)	2.153	1.535–3.019	<0.001	1.552	1.087–2.215	0.016
Risk score (High/Low)	2.718	1.957–3.776	<0.001	2.622	1.737–3.960	<0.001

*HR, hazard ratio; CI, confidence interval*.

### Evaluation of Immune Cell Infiltration

**Figure 8:**
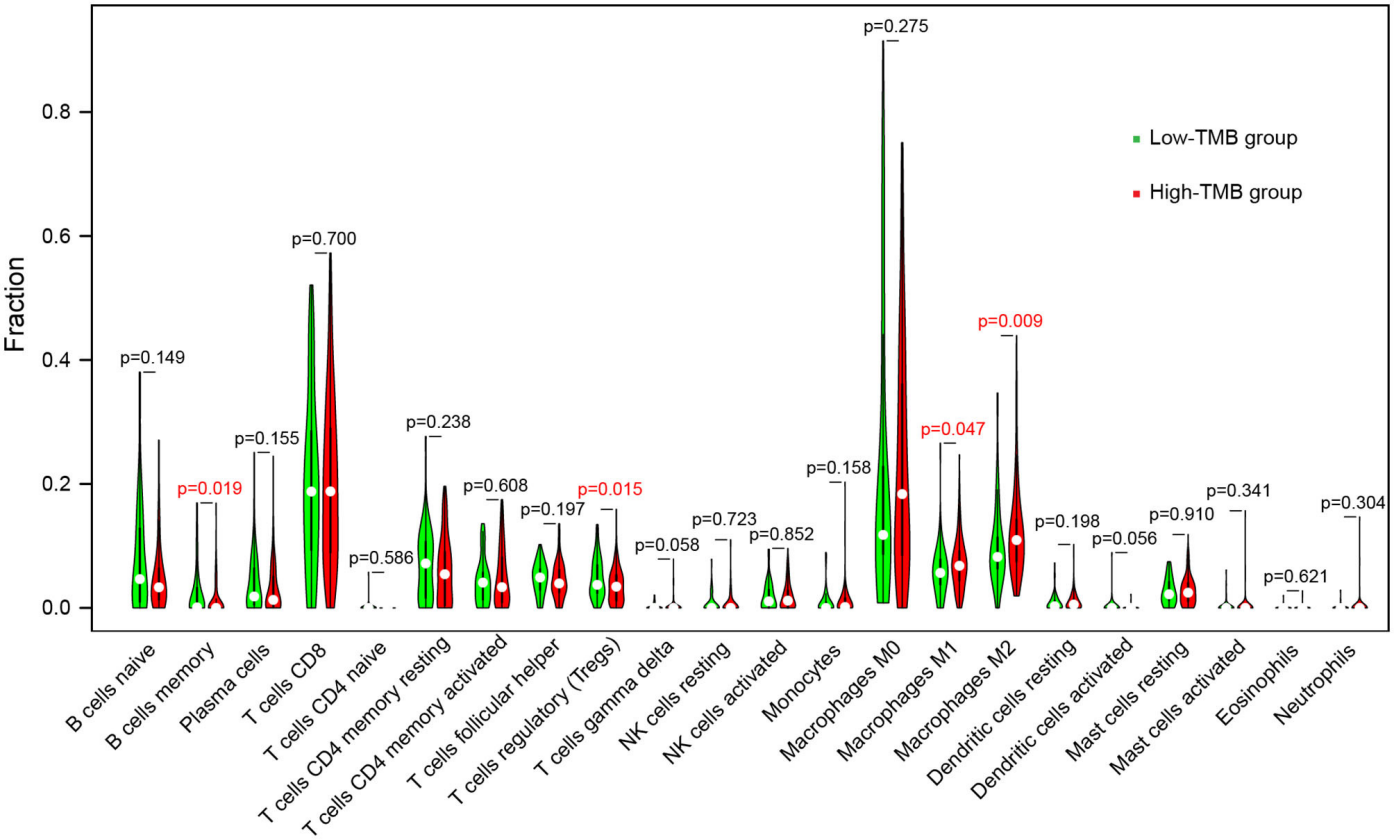
Comparisons of abundance of infiltrating immune cells between low-TMB and high-TMB groups.

To further explore the effect of TMB and prognostic modeling genes on immune cell infiltration, we calculated the distribution of 22 types of infiltrating immune cells in 449 melanoma samples based on the CIBERSORT algorithm (Supplementary Table 3). With *P* < 0.05 as the filter condition, the proportion of immune cells in 184 samples were exhibited in a barplot (Supplementary Figure 4). Then, for each type of immune cell, we compared the difference of proportion between high- and low-TMB groups using the Wilcoxon rank-sum test and visualized the results in a violin plot. Samples with higher TMB level had a significant decrease in the fraction of memory B cells (*P* = 0.019) and regulatory T cells (Tregs) (*P* = 0.015), and a significant increase in the fraction of macrophages M1 (*P* = 0.047) and macrophage M2 (*P* = 0.009) (Figure 8).

**Figure 9:**
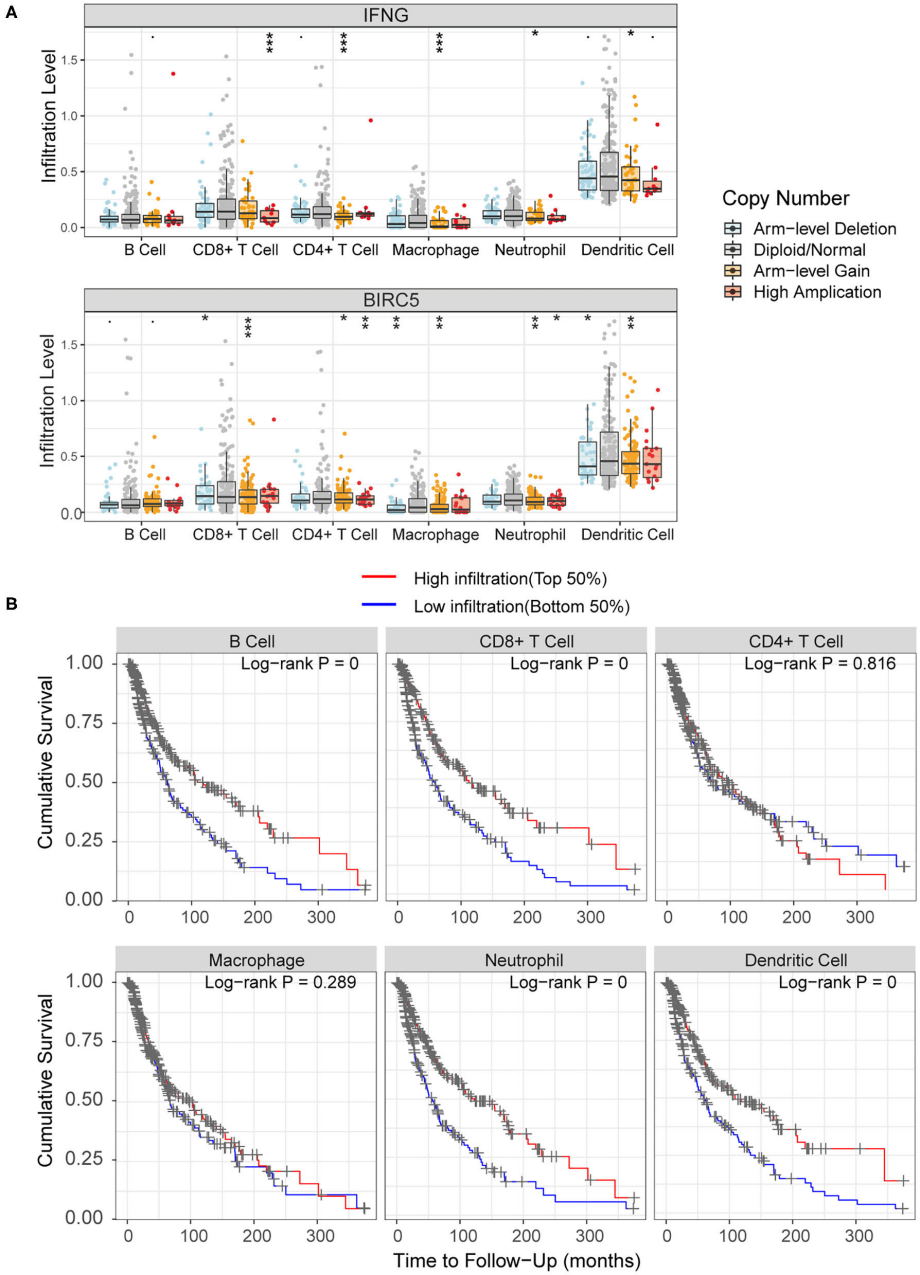
Association of immune infiltration with hub genes and prognosis. **(A)** Comparisons of immune infiltration between sample with copy number alteration and diploid of *IFNG* and *BIRC5*. **(B)** High infiltration level of B cell, CD8+ T cell, neutrophil and dendritic cell were associated with better survival outcomes.·*P* < 0.01; *P < 0.05; **P < 0.01; ***P < 0.001.

As for the relationship between prognosis-related genes and immune cell infiltration, we explored the changes of infiltration in the samples with copy number alteration of *IFNG* and *BIRC5*, respectively. Overall, compared to melanoma samples with diploid/normal expression of *IFNG* and *BIRC5*, samples with bidirectional copy number variation of *BIRC5* and increased copy number variation of *IFNG* had a lower level of immune infiltration, including B cells, CD4+ T cells, CD8+ T cells, macrophages, neutrophils, and dendritic cells (Figure 9A). Furthermore, we used the abundance of six immune cells and the expression level of two prognostic modeling genes to construct a Cox regression model. Cox analysis implied that higher levels of CD4+ T cell (HR = 20.246, *P* = 0.049), macrophage (HR = 12.960, *P* = 0.033), *BIRC5* expression (HR = 1.321, *P* = 0.001) and lower levels of neutrophil (HR < 0.001, *P* = 0.020) were risk factors for prognosis in melanoma patients (Table 4). Perhaps the expression of *IFNG* was correlated with the abundance of immune cells, so it was regarded as a confounding variable. Then we performed K–M analyses on six immune cells and revealed that higher infiltration levels of B cell, CD8+ T cell, neutrophil, and dendritic cell were associated with better survival outcomes (Figure 9B).

**Table 4:** Multivariate Cox regression analyses of immune cells and hub genes with OS.

**Variables**	**Coef**	**HR**	**95% CI**	***P-*value**
B cell	−1.934	0.145	0.004–5.234	0.291
CD8+ T cell	0.218	1.243	0.091–16.902	0.870
CD4+ T cell	3.008	20.246	1.012–404.962	0.049
Macrophage	2.562	12.960	1.232–136.312	0.033
Neutrophil	−7.836	0.000	0.000–0.285	0.020
Dendritic	−0.757	0.469	0.080–2.768	0.403
IFNG	−0.101	0.904	0.665–1.227	0.516
BIRC5	0.278	1.321	1.125–1.551	0.001

*Coef, coefficients; HR, hazard ratio; CI, confidence interval*.

## Discussion

Melanoma is one of the most aggressive forms of skin cancer, it accounts for only 5% of all skin cancer cases, but 80% of all skin cancer deaths (44). The occurrence, development, and evolution of melanoma are based on the accumulation of genomic changes, including high ultraviolet-driven mutation burdens, which makes melanoma the most immunogenic tumor (45, 46). Therefore, for melanoma patients, immunotherapy is used as adjuvant therapy after surgical resection in AJCC stage III melanoma, as well as in unresectable and metastatic cases (47). However, not all patients respond well to immunotherapy, thus biomarkers that predict treatment response are necessary to optimize patient benefit. According to previous studies, TMB and immune cell infiltration are both predictors of response to immunotherapy, but each has its own limitations (48–51). Consequently, further study of the association may help to identify hub genes and critical functional pathways, thereby constructing a more accurate combined biomarker model to predict the response in melanoma.

In the current study, we analyzed the somatic mutation profiles in cutaneous melanoma samples. The C > T mutations accounted for the vast majority, consistent with ultraviolet exposure leading to the formation of pyrimidine dimers (52). The three most frequently mutated genes were *TTN* (72%), *MUC16* (67%), *BRAF* (51%). *TTN*, mutations of which are often detected in solid tumors, is associated with increased TMB and better response to ICIs, and patients with mutant *TTN* have a better prognosis (53). *MUC16*, the coding gene of mucin 16, promotes the proliferation and metastasis of cancer cells and may also have immunosuppressive effects (54, 55). Meanwhile, cancer antigen 125 (CA125), as an epitope present on mucin16, is the most famous biomarker to monitor the serous ovarian cancer (56). The *BRAF* mutation is obviously the most common carcinogenic driver in melanoma, by activating the mitogen-activated protein kinase (MAPK) pathway, which is a pivotal regulator of cellular growth and proliferation (57–59).

Based on the mutation profiles, the correlation of TMB with prognosis and clinical features was further analyzed. Patients in the high-TMB group had significantly better survival outcomes. In previous studies, even without immunotherapy, higher TMB represented a better prognosis from adjuvant chemotherapy in patients with colorectal cancer and resected non-small-cell lung cancer (60, 61), but the correlation was not significant in melanoma (62). Besides, our research found that older patients and male patients have higher TMB levels, which is consistent with the significant trend of TMB increasing with age, with a 2.4-fold difference between age 90 and age 10 years (63). As for the difference between genders, perhaps due to the poor ability of men to clear the mutation-rich population of tumor cells, resulting in the accumulation of TMB (64).

TMB-related DEGs were identified, and the functional enrichment analysis and PPI analysis revealed that these DEGs were mainly associated with immune-related and cell adhesion-related pathways. It can be found that TMB is closely related to tumor immune infiltration and tumor microenvironment. Moreover, abnormal adhesion of tumor cells is associated with tumor progression and metastasis (65). In addition to the above, DEGs were also related to pathways such as osteoclast differentiation, which is involved in the bone metastasis of breast cancer (66, 67), TNF signaling pathway, and Wnt signaling pathway, which are related to the progression and metastasis of melanoma (68, 69).

The risk score model was constructed based on the expression level of two genes, *IFNG* and *BIRC5*. *IFNG* played a protective role, while *BIRC5* increased the risk. According to the AUCs of ROC curves in the TCGA cohort and three validation sets from the GEO database, the model exhibited a relatively good predictive accuracy, but there was still room for improvement, thus need further confirmation and modification in larger sample researches.

*IFNG* is the coding gene of interferon-gamma (IFN-γ). IFN-γ is a cytokine, which is critical for promoting immune response and anti-tumor immunity (70). As targets of ICIs, higher expression levels of PD-L1 and CTLA-4 in melanoma often represent better clinical response and therapeutic efficacy (71, 72). Moreover, the expression of PD-L1 can be upregulated by IFN-γ, and the absence of IFN-γ signaling pathway in tumor cells leads to the resistance to CTLA-4 targeting therapy, so that IFN-γ is a novel biomarker to predict the response to ICIs (73, 74). Perhaps the expression of *IFNG* can be combined with TMB and PD-L1 to construct a more accurate prediction model for immunotherapy in melanoma.

*BIRC5* encodes a survivin protein that belongs to a class of inhibitors of apoptosis protein, which is critical in the regulation of apoptosis and mitosis (75, 76). The *BIRC5* is rarely expressed in normal tissues but overexpressed in most types of tumors including melanoma, and the expression level is correlated with aggressive disease progression and poor clinical outcomes (77–79). Therefore, *BIRC5* and survivin are considered as tumor diagnostic and prognostic biomarkers, and inhibitors and immunotherapies targeting them have been developed (80, 81).

Based on the risk score model and clinical features, a nomogram was performed to predict the survival possibility in melanoma. Among the significant factors, in addition to age and pathological stage, there is a specific predictor of melanoma, ulceration indicator, which is a major prognostic factor according to the AJCC melanoma staging system (82). Although the C index of 0.702 and calibration curves exhibited relatively accurate prediction ability, further modifications and improvements are still necessary based on researches with more complete clinical information.

The association of immune cell infiltration with TMB was explored. The proportion of macrophages M1 and macrophages M2 in the high-TMB group was higher, while memory B cells and Tregs abundance in the low-TMB group was higher. The function of macrophages in the tumor is complex and two-sided. Macrophages M1 initiate the production of cytokines in the tumor microenvironment and promote the destruction of tumor cells (83), while macrophages M2, especially the tumor-associated macrophages (TAMs), play an important role in tumor growth and metastasis (84, 85). TAMs provide a promising target for immunotherapy, and TAMs targeting can enhance the response to other immunotherapies when used synergistically (86, 87). Tregs maintain the immune homeostasis via suppressing the immune response and inhibit the anti-tumor effect in the tumor microenvironment (88). Therefore, Tregs targeted immunotherapy, such as the depletion of Tregs, can enhance the therapeutic efficacy of ICIs (89).

Moreover, according to survival analyses, higher infiltration levels of B cell, CD8+ T cell, neutrophil, and dendritic cell represented better survival outcomes. Tumor infiltrating B cells play a critical role in regulating the anti-tumor immune response in melanoma, and the absence of B cells is associated with a poor response to ICIs (90, 91). CD8+ T cells constitute an important part of the immune response to tumors and play a critical role in killing tumor cells (92). The abundance of CD8+ tumor infiltrating cells is positively correlated with the prognosis of patients with melanoma (93). Dendritic cells are involved in the processing and presentation of tumor antigens to naive T cells, which then stimulate T cell proliferation and induce the specific immune responses (94). Recently, tumor vaccines based on dendritic cells have gradually become the focus of research and has been used for the clinical treatment in melanoma (95). As for neutrophil, although there are relatively few studies on neutrophils alone, the neutrophil-to-lymphocyte ratio (NLR) is regarded as a novel biomarker, and a lower NLR is associated with better prognosis, better response to ICIs, and less recurrence in melanoma (96–98).

However, there are still some limitations in the current study that must be considered. In further studies, a large sample clinical cohort is required to verify the impact of TMB on prognosis, the accuracy of the risk score model, and nomogram. Moreover, basic experiments are required to verify the relationship between TMB and immune infiltration.

## Conclusions

In cutaneous melanoma, higher TMB was associated with better survival outcomes. TMB-related DEGs were mainly involved in immune-related and cell adhesion-related pathways. The risk score model and nomogram had relatively high predictive capability on survival outcomes. The relationship between TMB and immune infiltration, especially the abundance of macrophages and Tregs, can provide a reference for further advanced prediction model of response to immunotherapy.

## Data Availability Statement

Publicly available datasets were analyzed in this study. This data can be found here: TCGA (https://portal.gdc.cancer.gov/), GEO (https://www.ncbi.nlm.nih.gov/geo/), ImmPort (https://www.immport.org/shared/genelists/), and TIMER (https://cistrome.shinyapps.io/timer/).

## Author Contributions

XW designed the research. KK, FX, and JM performed the bioinformatics analyses and wrote the manuscript. YB downloaded and managed the genomic, gene expression, and clinical data from public databases and participated in the discussion. All authors read and approved the final version of the manuscript. All authors reviewed and revised the manuscript.

## Conflict of Interest

The authors declare that the research was conducted in the absence of any commercial or financial relationships that could be construed as a potential conflict of interest.
