# Supplementary material for: Significance of Tumor Mutation Burden in Immune Infiltration and Prognosis in Cutaneous Melanoma
Source: Front Oncol. 2020 Sep 18;10:573141. doi: 10.3389/fonc.2020.573141 (PMC7531222; doi:10.3389/fonc.2020.573141)
Supplement: Supplementary file 4 [file Data_Sheet_1.docx]

Supplementary Material

# Supplementary Figures and Tables

## Supplementary Figures

**
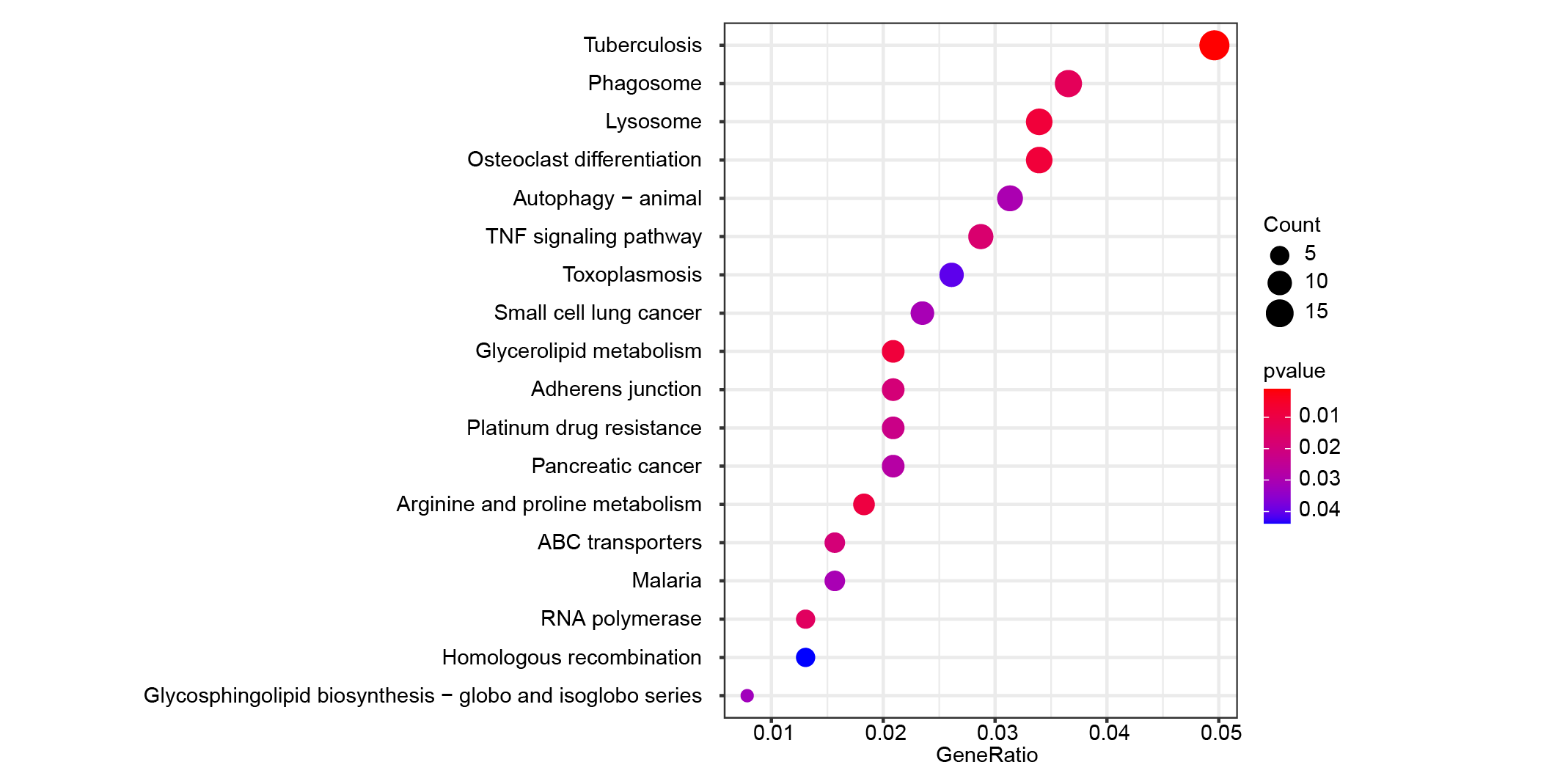
**

**Supplementary Figure 1.** KEGG enrichment analysis of DEGs.

**
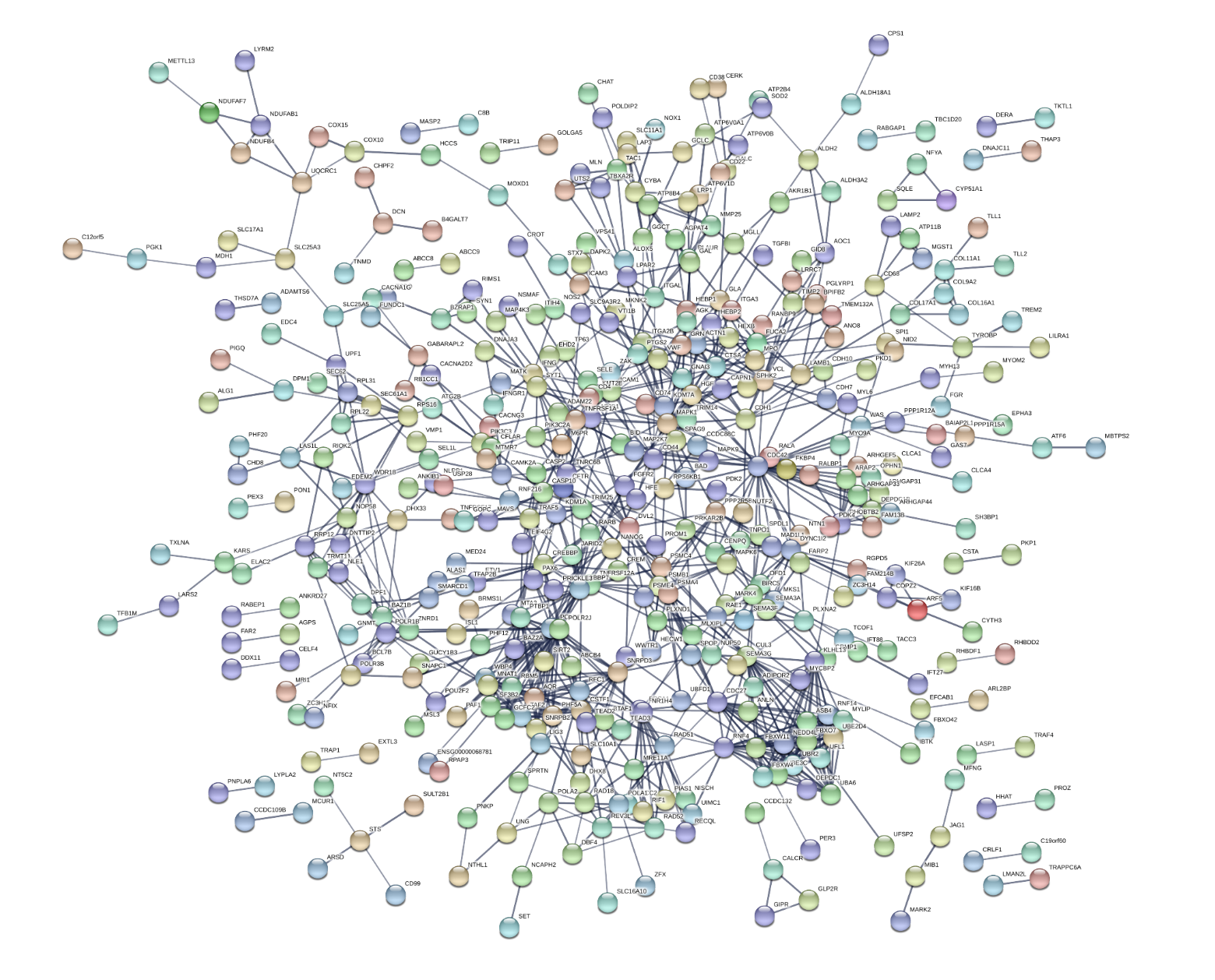
**

**Supplementary Figure 2.** PPI network of DEGs constructed by STRING database.

**
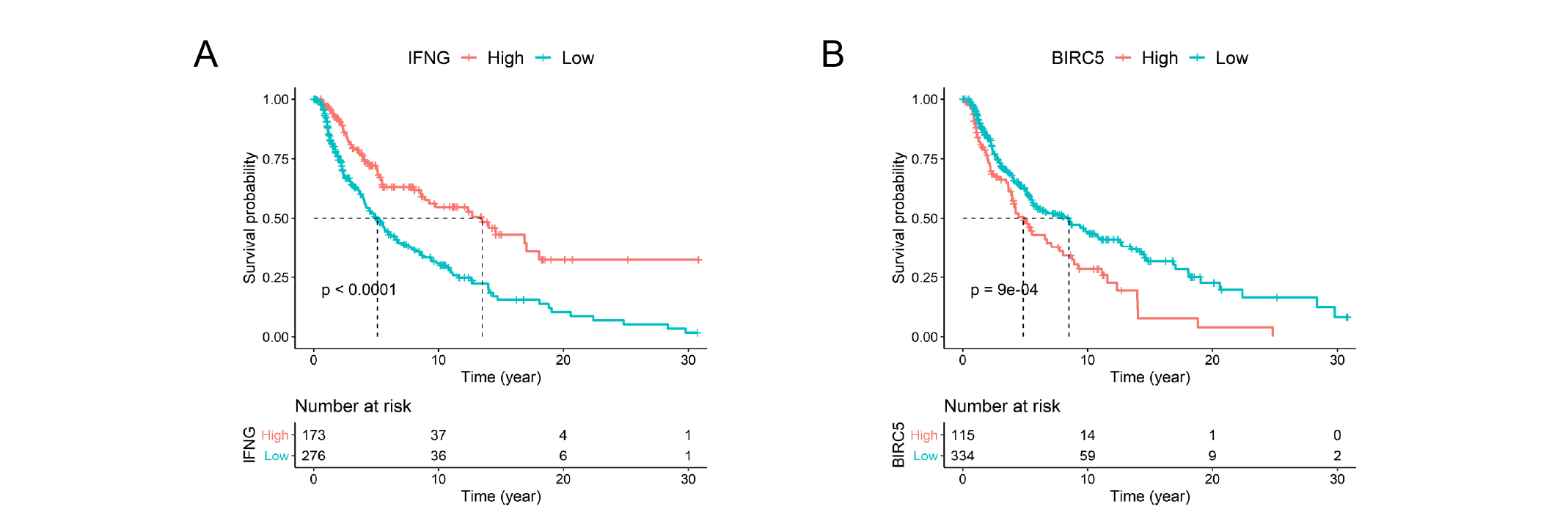
**

**Supplementary Figure 3.** Higher expression level of *IFNG* represented better survival outcomes, while *BIRC5* on the contrary.


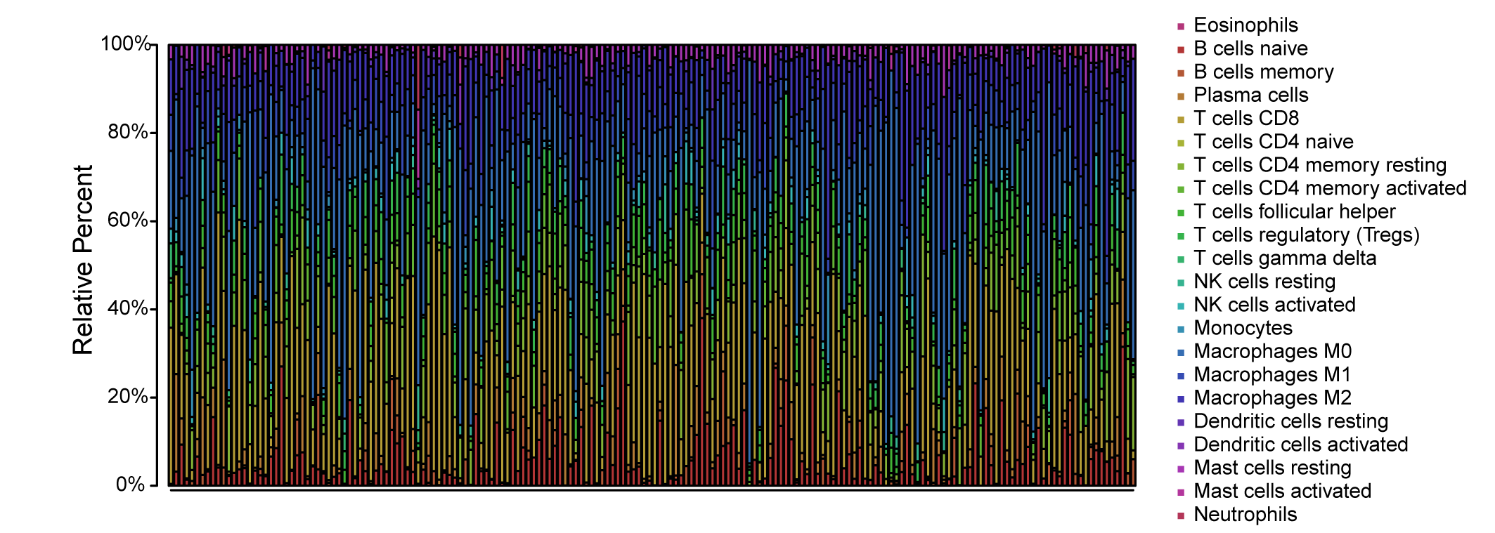


**Supplementary Figure 4.** The fractions of 22 immune cells infiltrated in each sample of melanoma.
